# Enhancement of Apx Toxin Production in *Actinobacillus pleuropneumoniae* Serotypes 1, 2, and 5 by Optimizing Culture Conditions

**DOI:** 10.4014/jmb.1912.12042

**Published:** 2020-03-27

**Authors:** Hoai Thu Dao, Van Tan Do, Quang Lam Truong, Tae-Wook Hahn

**Affiliations:** 1 College of Veterinary Medicine and Institute of Veterinary Science, Kangwon National University, Chuncheon 24341, Republic of Korea; 2 Key Laboratory of Veterinary Biotechnology, Faculty of Veterinary Medicine, Vietnam National University of Agriculture, Hanoi, Vietnam; 3 Innovac Co., Kangwon National University, 1 Kangwondaehak-gil, Chuncheon 24341, Republic of Korea

**Keywords:** *Actinobacillus pleuropneumoniae*, Apx toxins, production yields

## Abstract

*Actinobacillus pleuropneumoniae* (APP) is a causative agent of porcine pleuropneumonia. Therefore, the development of an effective vaccine for APP is necessary. Here, we optimized the culture medium and conditions to enhance the production yields of Apx toxins in APP serotype 1, 2, and 5 cultures. The use of Mycoplasma Broth Base (PPLO) medium improved both the quantity and quality of the harvested Apx toxins compared with Columbia Broth medium. Calcium chloride (CaCl_2_) was first demonstrated as a stimulation factor for the production of Apx toxins in APP serotype 2 cultures. Cultivation of APP serotype 2 in PPLO medium supplemented with 10 µg/ml of nicotinamide adenine dinucleotide (NAD) and 20 mM CaCl_2_ yielded the highest levels of Apx toxins. These findings suggest that the optimization of the culture medium and conditions increases the concentration of Apx toxins in the supernatants of APP serotype 1, 2, and 5 cultures and may be applied for the development of vaccines against APP infection.

## Introduction

*Actinobacillus pleuropneumoniae* (APP) is a causative agent of porcine pleuropneumonia, which can cause sudden death in pigs in peracute form, leading to significant economic loss within the worldwide swine industry [1]. Vaccination is one of the most promising approaches to control and prevent porcine pleuropneumonia in pigs [2, 3]. Currently, the main commercial vaccines are bacterin vaccines, subunit vaccines, and toxoid vaccines [1]. However, 18 serotypes of APP have been identified [4], which contributes to the difficulty in combating the disease effectively.

The main virulence factors of APP include the outer membrane, capsule, repeats-in-toxin toxins, proteases, lipopolysaccharides, adhesins, and transferrin-binding proteins [5]. The virulence of APP is highly variable and depends on the serotype, which is closely correlated with the type of Apx exotoxins secreted [6]. Four types of Apx exotoxins have been identified and exhibit different functions and virulence [1, 7]. The ApxI toxin (105 kDa) is strongly hemolytic and strongly cytotoxic; the ApxII toxin (103 kDa) is weakly hemolytic and moderately cytotoxic, while the ApxIII toxin (120 kDa) is not hemolytic but strongly cytotoxic. ApxI, II, and III are considered as the most important virulence factors of APP and can be produced and secreted into culture supernatants. ApxIV (200 kDa) is present in all serovars of APP in natural infection [1].

Apx toxins play important roles in helping APP avoid the host clearance mechanisms, thus inducing lesions, and are highly immunogenic [8]. Therefore, Apx toxins are considered as the most important components and are used widely in the current commercial vaccines [1]. A vaccine comprising ApxI, II, and III and the 42 kDa outer- membrane protein showed good results in decreasing lethality and pathology in pigs against a virulent APP challenge [9]. Although several reports have provided methods of production of Apx toxins from APPs, there is a need for approaches that afford improvement of the production yields of these toxins. In this study, we optimized the culture medium and conditions to increase the production yield of Apx toxins from APP serotypes 1, 2, and 5.

## Materials and Methods

### Strains and Growth Conditions

The APP serotypes 1, 2, and 5 were isolated from the lungs of pigs with contagious pleuropneumonia in Korea and were stored at –70°C. The primers used in this study for identifying and serotyping APP are listed in Table 1. These primers were synthesized by Cosmogenetech (Korea). The APP strains were identified via nested PCR using primer pairs P1, P2, P3, and P4 (Table 1), as described previously [10]. Subsequently, multiplex PCR in a total of 20 µl was used for serotyping [11-13]. Briefly, 5 µl of the suspended colony was mixed into the PCR pre- mix containing 2× TOPsimple DyeMIX-Tenuto (Enzynomics, Korea) and 10 pmol of each primer (P5, P6, P7, P8, P9, and P10) (Table 1). The thermal cycling conditions included an initial denaturation at 95°C for 5 min, followed by 35 cycles of 95°C for 1 min, 55°C for 1 min, 72°C for 1 min 50 s; and a final extension at 72°C for 10 min [11].

The APP isolates were grown in nutrient medium supplemented with 10 µg/ml of nicotinamide adenine dinucleotide (NAD; Germany) at 37°C with shaking at 180 rpm.

### Growth Curves of APPs

To determine the optimal time point for harvesting Apx exotoxins, the growth curves of APP serotypes 1, 2, and 5 in Mycoplasma Broth Base (PPLO; Oxoid Ltd., UK) or Columbia Broth (CB; Difco BD, USA) medium supplemented with 10 µg/ml of NAD were determined within 8 h of inoculation. Optical density at 600 nm (OD_600nm_) was used to measure the density of cultures hourly.

### Optimization of Medium Used for Apx Toxin Production

To optimize the culture medium used for the production of a higher concentration of Apx toxins, PPLO or CB medium supplemented with 10 µg/ml of NAD was used to culture APP serotypes 1, 2, and 5. The cultures were stopped after 6 h of cultivation at 37°C and 5% CO_2_, and Apx toxins were harvested using the precipitation method, as described below.

### Optimization of CaCl_2_ Concentration for Apx Toxin Production

In previous studies, calcium chloride (CaCl_2_) was applied to increase the concentration of Apx toxins in APP serotype 1 and 5 cultures [14]. Here, we optimized the concentration of the CaCl_2_ supplement in APP serotype 2 cultures. A gradient of concentration ranging from 0 to 50 mM CaCl_2_ (Duchefa Biochemie B.V., The Netherlands) was applied to the cultures and the amount of Apx toxins was screened using SDS–PAGE.

### Collection of Apx Toxins Using the Precipitation Method

Apx exotoxins were prepared from APP cultures according to the methods reported previously [15, 16]. Cells were grown to the mid-log phase in the selected media containing 10 µg/ml of NAD and 20 mM CaCl_2_. The culture supernatants were collected using centrifugation at 8,000 ×*g* for 30 min at 4°C and mixed with 55% ammonium sulfate (Duchefa Biochemie B.V.), followed by incubation at 4°C overnight. The precipitated toxins were pelleted at 8,000 ×*g* for 30 min at 4°C, dissolved in 10 mM Tris-HCl (pH 7.5) [16] at a ratio of 1/85 of the original culture volume, and dialyzed in the same buffer at 4°C overnight. The collected toxins were stored at –70°C until use.

### Collection of Apx Toxins Using the Ultrafiltration Method

To compare the efficacy of the precipitation and ultrafiltration methods of harvesting Apx toxins from APP serotype 2 cultures, the APP serotype 2 was inoculated in PPLO medium supplemented with 10 µg/ml of NAD and 20 mM CaCl_2_ for 6 h at 37°C with shaking at 180 rpm. For precipitation, Apx toxins were collected as described above. For ultrafiltration, the culture supernatants were collected using centrifugation at 8,000 ×*g* for 30 min at 4°C, and Apx toxins were concentrated 85-fold by ultrafiltration using Amicon Ultra-15 Centrifugal Filters (Merck, Ireland) at 5,000 ×*g* for 10 min at 4°C and stored at –70°C until use.

### SDS–PAGE

To evaluate the expression ability and purity of Apx toxins, the collected toxins were separated by 12% SDS– PAGE and stained with Coomassie Brilliant Blue R-250 (Bio-Rad Laboratories Ltd., UK). To measure the concentration of the produced Apx toxins, the intensity of the target bands was compared with the intensity of the bands corresponding to the bovine serum albumin (BSA) standards (Thermo Fisher Scientific, USA) that were loaded onto the same gel, and concentrations were estimated based on the computerized arbitrary units [17] using the Gel Doc XR+ Imaging System (Bio-Rad Laboratories, USA).

### Statistical Analysis

Statistical analyses were performed and graphs were generated using the GraphPad Prism 5 program. Statistically significant differences are indicated as follows: *p < 0.05, **p < 0.01, and ***p < 0.001.

## Results

### Identification and Serotyping of APPs

**Fig. 1:**
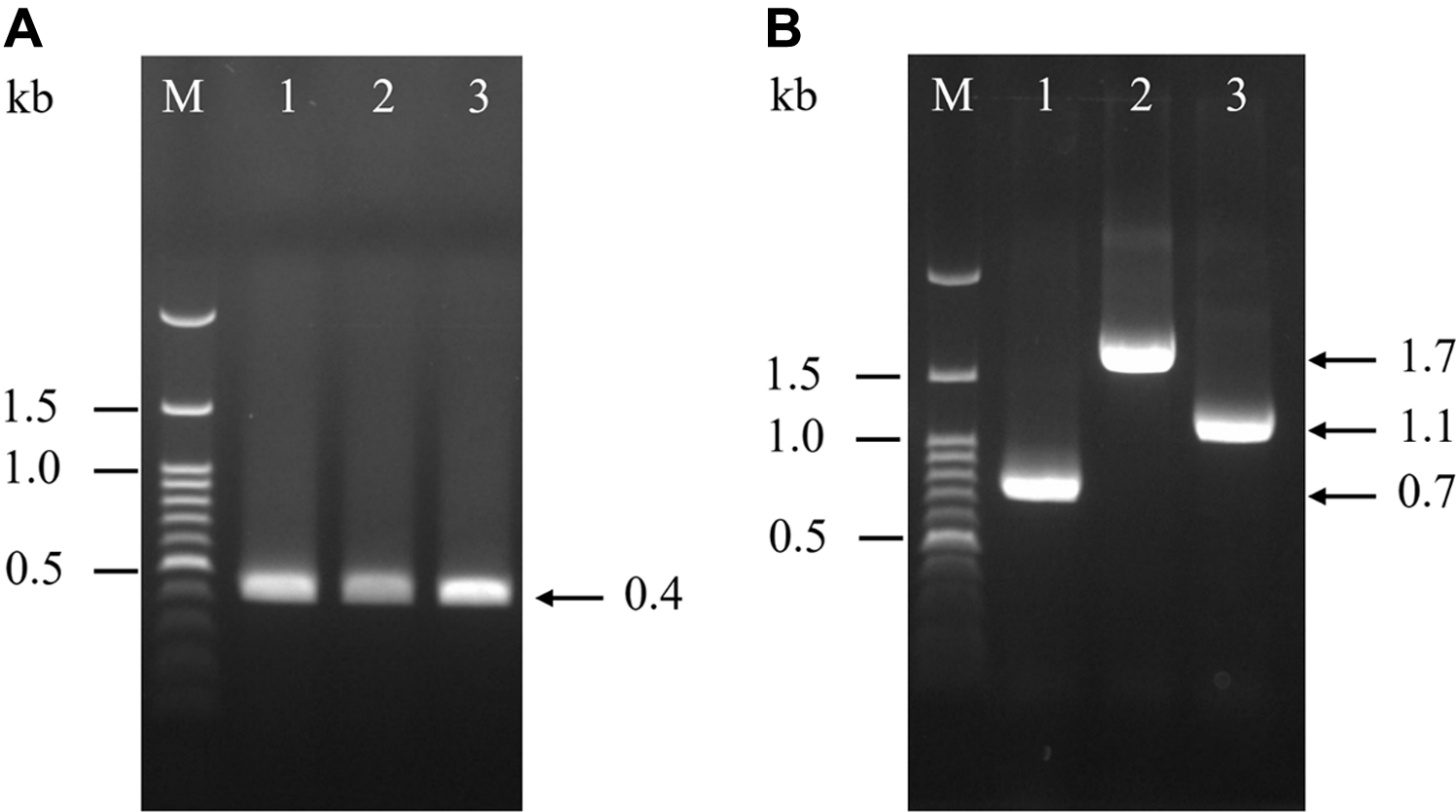
PCR for identifying (A) and serotyping (B) APP serotypes 1, 2, and 5. M, marker; lane 1, APP serotype 1; lane 2, APP serotype 2; and lane 3, APP serotype 5.

The APP serotypes were determined according to the criteria described in previous reports [10-13]. First, the APP isolates were confirmed as a band of 0.4 kb by nested PCR (Fig. 1A), followed by serotyping as APP serotypes 1, 2, and 5 corresponding to bands of 0.7, 1.7, and 1.1 kb, respectively (Fig. 1B).

### Optimization of Medium Used for Production of Apx Toxins

In previous studies, CB was used for cultivating and producing Apx toxins from APP serotypes 1, 2, and 5 [16, 18, 19]. In this study, we compared the production of Apx toxins in APP cultures using PPLO and CB media.

**Fig. 2:**
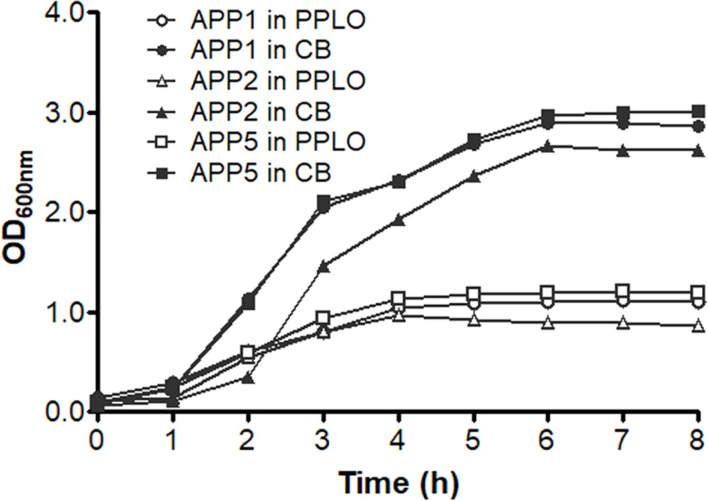
Growth curves of APP serotypes 1, 2, and 5 in PPLO and CB media containing 10 μg/ml NAD within 8h of cultivation.

To determine the optimal time point for harvesting Apx toxins from APP cultures, growth curves of APP serotypes 1, 2, and 5 in PPLO or CB medium containing 10 µg/ml of NAD were prepared. APP cultures reached the stationary phase at 6 h of inoculation for all three APP serotypes (Fig. 2). Therefore, this time point was chosen for harvesting Apx toxins in subsequent experiments.

**Fig. 3:**
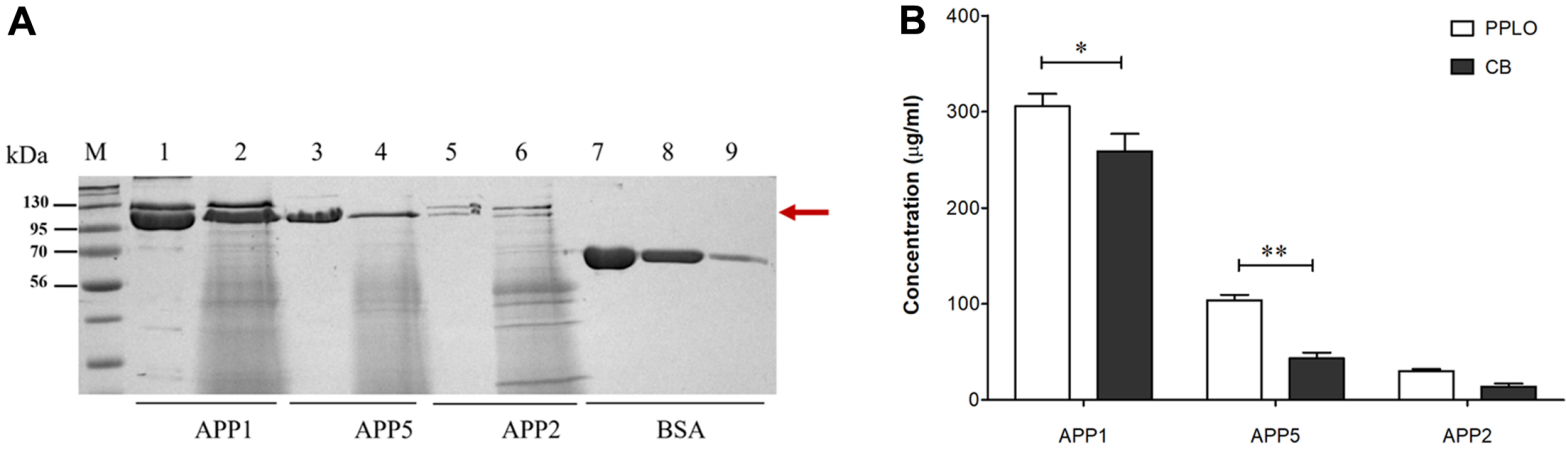
Optimization of the medium used for the production of Apx toxins in APP serotype 1, 2, and 5 cultures (**A**) SDS–PAGE analysis of the Apx toxins secreted from APP serotypes 1, 2, and 5 in PPLO and CB media containing 10 μg/ml NAD. M, protein marker; lanes 1, 3, and 5, Apx toxins in the PPLO medium; lanes 2, 4, and 6, Apx toxins in the CB medium; lanes 7, 8, 9, BSA standards (250, 125, and 25 μg/ml, respectively). (**B**) The relative intensity of the SDS–PAGE bands illustrates the Apx toxin production yields. The asterisks indicate significant differences (*p < 0.05; **p < 0.01).

As shown in Fig. 3, the PPLO medium supplemented with 10 µg/ml of NAD induced the production of a higher level of Apx toxins compared with the CB medium containing 10 µg/ml of NAD. For APP serotype 1, the concentration of Apx toxins harvested from the culture using the PPLO medium was significantly higher than that using the CB medium (mean, 305 and 258 µg/ml, respectively; *p* < 0.05) (Fig. 3B). In addition, the levels of Apx toxins collected from cultures of APP serotypes 2 and 5 were doubled in the PPLO medium compared with the CB medium (Fig. 3B), indicating that the PPLO medium had a positive effect on the production of Apx toxins. Furthermore, the Apx toxins produced by the three APP serotypes in the PPLO medium yielded a lower number of nonspecific bands and a clearer background on SDS–PAGE (Fig. 3A). In contrast, Apx toxins recovered from APP serotypes 1, 2, and 5 cultured in the CB medium exhibited numerous bands and a darker background. Collectively, these findings demonstrated that the PPLO medium supplemented with 10 μg/ml NAD is more appropriate for the production of Apx toxins from APP serotype 1, 2, and 5 cultures.

### Optimization of CaCl_2_ Concentration for Production of Apx Toxins

Van Den (1997), Haga *et al.* (2007), and Frey and Nicolet (1988) established the concentration of CaCl_2_ in APP serotype 1 and 5 cultures [16, 18, 19]. In this study, we optimized the concentration of CaCl_2_ in PPLO medium with 10 µg/ml of NAD to increase the amount of Apx toxins produced in APP serotype 2 cultures.

**Fig. 4:**
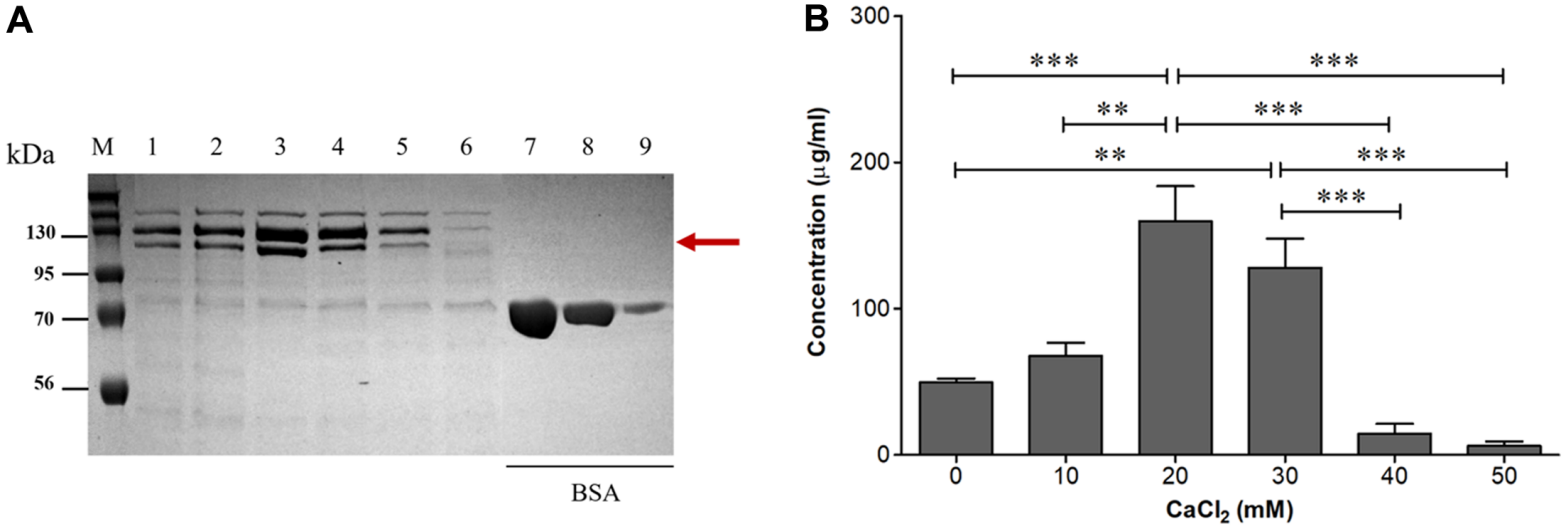
Optimization of the CaCl_2_ concentration used for supplementation of APP serotype 2 cultures. (**A**) SDS–PAGE analysis of the Apx toxins secreted in APP serotype 2 cultures using PPLO medium containing 10 μg/ml NAD and different concentrations of CaCl_2_. M, protein marker; lanes 1–6, the medium supplemented with 0–50 mM CaCl_2_, respectively; lanes 7, 8, 9, BSA standards (250, 125, and 25 μg/ml, respectively). (**B**) The relative intensity of the SDS–PAGE bands illustrates the Apx toxin production yields. The asterisks indicate significant differences (**p < 0.01; ***p < 0.001).

The results obtained showed that dose-dependent changes in the concentration of Apx toxins accompanied the variation in the concentrations of CaCl_2_ in the culture medium (Fig. 4). CaCl_2_ supplementation at 10, 20, and 30 mM increased the levels of Apx toxins in the cultures. The production yields of Apx toxins were significantly higher in the medium containing 20 mM (*p* < 0.001) and 30 mM (*p* < 0.01) CaCl_2_ than they were in other cultures and in cultures without CaCl_2_, and reached a peak (160 µg/ml) at 20 mM CaCl_2_. In contrast, higher concentrations of CaCl_2_ appeared to have a negative effect on the production yields. Concentrations of CaCl_2_ of 40 and 50 mM inhibited the expression of Apx toxins dramatically compared with 20 mM CaCl_2_ (*p* < 0.001), to a level that was lower than that observed in cultures performed in PPLO medium without CaCl_2_. Taken together, these results indicate that PPLO medium supplemented with 10 µg/ml of NAD and 20 mM CaCl_2_ stimulates effectively the secretion of Apx toxins from APP serotype 2.

### Selection of a Collection Method for Apx Toxins

**Fig. 5:**
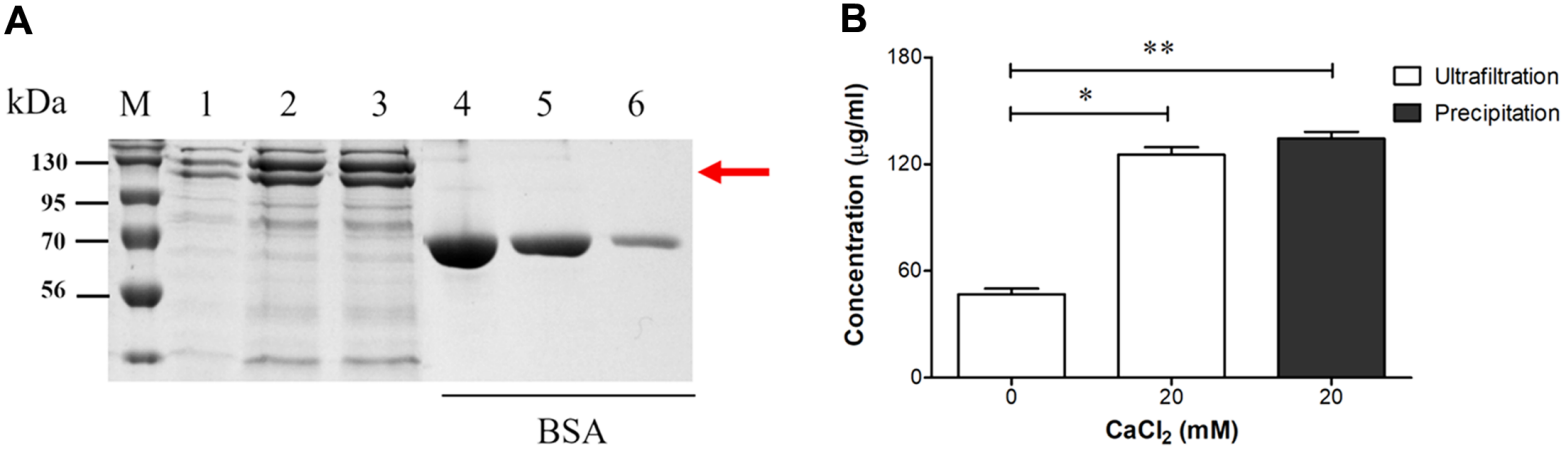
Optimization of the harvesting method of Apx toxins in APP serotype 2 cultures. (**A**) SDS–PAGE analysis of the concentration of Apx toxins using different harvesting methods. M, protein marker; lane 1, PPLO medium containing 10 μg/ml NAD without CaCl_2_, ultrafiltration method; lane 2, PPLO medium containing 10 μg/ml NAD and 20 mM CaCl_2_, ultrafiltration method; lane 3, PPLO medium containing 10 μg/ml NAD and 20 mM CaCl_2_, precipitation method; lanes 4, 5, 6, BSA standards (250, 125, and 25 μg/ml, respectively). (**B**) The relative intensity of the SDS–PAGE bands illustrates the Apx toxin production yields. The asterisks indicate significant differences (*p < 0.05; **p < 0.01).

The concentration of CaCl_2_ was optimized to 20 mM, as it produced the highest level of Apx toxins. We compared the efficacy of the ultrafiltration and precipitation methods for harvesting Apx toxins in APP serotype 2 cultures. As shown in Fig. 5, the concentration of Apx toxins that were collected using the precipitation method was slightly higher than that obtained using the ultrafiltration method (mean, 134 and 125 µg/ml, respectively); however, this difference was not significant (*p* > 0.05). This result suggests that the two purification methods can be applied to the collection of Apx toxins secreted from APP serotype 2.

## Discussion

APP serotypes 1 and 5 are predominant in Korea [20]. They are the most virulent serotypes and produce both ApxI and ApxII toxins [21, 22]. Moreover, the less virulent APP serotype 2, which produces ApxII and ApxIII toxins, also contributes to the infection of pigs in Korea [20]. Because of their prevalence and Apx toxin production patterns, these serotypes are used widely in the development of bacterins and Apx toxoid vaccines in Korea. Therefore, we optimized the culture conditions to improve the production yields of Apx toxins in APP serotype 1, 2, and 5 cultures.

In previous studies, CB, PPLO, tryptic soy broth (TSB), and brain heart infusion (BHI) media have been used for the cultivation of APP [18,19,23-25]. However, TSB and BHI media cause precipitation when supplemented with 20 mM CaCl_2_ for the enhancement of Apx toxin production. In contrast, CB and PPLO media do not cause precipitation after the addition of CaCl_2_; hence, they are suitable for Apx toxin production.

In this study, we described the process used to harvest high concentrations of Apx toxins from APP serotype 1, 2, and 5 cultures. The growth curves of APP serotypes 1, 2, and 5 in PPLO or CB media containing 10 µg/ml of NAD were determined (Fig. 2). It has been reported that the amount of Apx toxins varies throughout the APP growth curve, and peaks at a high APP cell density (from the late logarithmic to the early stationary phase) [26]. Therefore, the harvesting time was set at 6 h of inoculation in this study, which was consistent with previous studies [15, 16, 18, 25, 27].

SDS–PAGE using BSA as a standard is a suitable method for the quantification of specific protein bands of interest, for the determination of the purity of proteins and standardization of concentrations among different toxin preparations [28, 29]. Hence, this method was used in several previous reports [17,28-30]. The target bands were confirmed as Apx toxins according to our previous study [31], in which the secretion genes were deleted, leading to the absence of the Apx toxin bands on SDS–PAGE. Frey and Nicolet (1988) also showed that precipitation using ammonium sulfate can separate the Apx toxins from several proteins [19]; thus, Apx toxins are predominant among the harvested proteins. These findings prove that the target bands exhibiting a molecular mass of 110–130 kDa on SDS–PAGE are Apx toxins.

Although numerous studies have used the CB medium to produce Apx toxins [15, 16, 18, 19, 32], the optimization of production of these toxins using the PPLO medium has not been reported. Here, CB and PPLO media with equally added 10 µg/ml of NAD were applied to the production of Apx toxins in APP serotype 1, 2, and 5 cultures (Figs. 3A and 3B). The levels of Apx toxins were significantly higher in the PPLO than those in the CB medium (Fig. 3B). The concentrations of Apx toxins obtained using the PPLO medium in this study were approximately 30, 100, and 300 µg/ml for APP serotypes 2, 5, and 1, respectively. In addition, the collected toxins yielded a clearer background on SDS–PAGE in the case of the PPLO medium (Fig. 3A). This result indicates that PPLO supplemented with 10 µg/ml of NAD is more appropriate for the production of Apx toxins from APP cultures, as it was more effective in the purification step and yielded fewer nonspecific components in the final products.

The Apx toxins contain glycine-rich nonapeptides that exhibit strong binding to Ca2+ cations [33]. Thus, Ca2+ plays an important role in the induction of the expression of Apx toxins in APP [14]. Supplementation with CaCl_2_ has been used to enhance the production of Apx toxins in the cultures of APP serotypes 1 and 5 [16, 18, 19, 26]. Here, we optimized CaCl_2_ supplementation in APP serotype 2 cultures. To our knowledge, this is the first investigation of the increase in the levels of Apx toxins in APP serotype 2 cultures afforded by CaCl_2_ supplementation. After 6 h of cultivation of APP serotype 2 in PPLO medium containing 10 µg/ml of NAD and a range of CaCl_2_ concentrations, the toxins were collected using the precipitation method and analyzed. The addition of CaCl_2_ significantly increased the production yields of Apx toxins in APP serotype 2 cultures. The highest toxin concentration (160 μg/ml) was observed after supplementation with 20 mM CaCl_2_. In contrast, a reversion was observed in the presence of supplementation with higher concentrations (40 and 50 mM) of CaCl_2_, at which the production of the toxins in APP serotype 2 cultures was even decreased (Figs. 4A and 4B). Our results are consistent with those of previous studies, in which CaCl_2_ concentrations of 10 to 25 mM were recommended for APP serotype 1 and 5 culture [15, 16, 18, 19]. This result suggests that PPLO medium containing 10 µg/ml of NAD and 20 mM CaCl_2_ effectively stimulates the expression of Apx toxins in APP serotype 2 cultures.

Ultrafiltration and precipitation have been applied for harvesting Apx toxins from APP cultures. We conducted the first comparison between the two methods in the context of Apx toxin production from APP serotype 2 cultures. A slightly higher level of Apx toxins was observed using the precipitation method in comparison with the ultrafiltration method; however, this difference was not significant (Figs. 5A and 5B). Collectively, the results of the present study suggest a significant improvement in the levels of Apx toxins collected from APP cultures using an optimized medium.

In conclusion, in this study, we described the optimization of the culture medium and conditions, and the toxin collection method to enhance the yield of Apx toxins in APP serotype 1, 2, and 5 cultures. In particular, the levels of Apx toxins in APP serotype 2 cultures were significantly increased using an optimized medium with an appropriate CaCl_2_ concentration and harvesting method. These findings may represent a good strategy for the development of toxoid vaccines or effective combination vaccines against APP infection.

## Figures and Tables

**Table 1:** Primers used in this study.

Primer	Sequence (5′→3′)	Target	PCR product (bp)	Reference
Primers used for identifying APP				
P1	TGGCACTGACGGTGATGA	*apx*IVA	442	[10]
P2	GGCCATCGACTCAACCAT			
P3	GGGGACGTAACTCGGTGATT	*apx*IVA	378	
P4	GCTCACCAACGTTTGCTCAT			
Primers used for serotyping APP				
P5	GGGCAAGCCTCTGCTCGTAA	APP1	754	[12]
P6	GAAAGAACCAAGCTCCTGCAAT			
P7	CGCAGCCGGACAAAAACAAATACACG	APP2	1724	[11]
P8	CACCCCATGAATCGACTGATTGCCAT			
P9	TTTATCACTATCACCGTCCACACCT	APP5	1107	[13]
P10	CATTCGGGTCTTGTGGCTACTAAA			
